# Effect of Salt Reduction on the Quality of Boneless Dry-Cured Ham from Iberian and White Commercially Crossed Pigs

**DOI:** 10.3390/foods11060812

**Published:** 2022-03-12

**Authors:** Beatriz Muñoz-Rosique, Eva Salazar, Julio Tapiador, Begoña Peinado, Luis Tejada

**Affiliations:** 1 Departamento de Calidad, Aromaibérica Serrana, S.L. Ctra. Fuente Álamo, km 17,4, 30332 Murcia, Spain; beatriz@aromais.es; 2 Departamento de Tecnología de la Alimentación y Nutrición, UCAM Universidad Católica de Murcia, Campus de los Jerónimos, 30107 Murcia, Spain; ltejada@ucam.edu; 3 Divisa Ibérica Plus S.L, 45519 Novés, Spain; juliotapiador@yahoo.es; 4 Instituto Murciano de Investigación y Desarrollo Agrario y Medioambiental (IMIDA), Equipo de Mejora Genética Animal, 30150 Murcia, Spain; begona.peinado@carm.es

**Keywords:** Iberian dry-cured ham, salt reduction, deboned ham, food labeling, proteolysis

## Abstract

Iberian dry-cured ham has great value in a traditional Spanish diet, although experts have recommended its consumption should be reduced because of its high salt content and link to cardiovascular diseases. Eighteen boneless Iberian hams (RIB), eighteen boneless white commercially crossed pig hams (RWC), and eighteen traditionally salted and processed Iberian hams (TIB) were manufactured to check whether the breed (RIB vs. RWC) or the processing (RIB vs. TIB) affects their physical–chemical and sensory characteristics. Moisture, protein, total nitrogen, nonprotein nitrogen, proteolysis index, NaCl, and ash contents were higher in RWC, contrary to the fat values, which were more than double in RIB. All macrominerals, except Ca, were affected by the processing stage and breed, whereas only the micromineral Zn was higher in RWC. The breed did not affect the free amino acid content; however, the total content was slightly higher in RWC. Regarding the manufacturing process, the deboning of RIB allowed the reduction of salt by over 30%. However, the microbiological stability was not affected, resulting in a safe product. Although deboning and salt reduction significantly affect the hardness, adhesiveness, deformation, and elasticity of dry-cured hams, consumers value all sensory parameters with higher scores in RIB.

## 1. Introduction

Iberian dry-cured ham is considered one of the important products of the Spanish meat industry. It is a quality product known worldwide, which has different organoleptic characteristics from other meat products, which make its consumption palatable. It is recognized for its high biological value because of its rich composition of proteins, unsaturated fatty acids, iron, zinc, and vitamin B, among others. In addition, it has a high economic value [1].

Despite having these nutritional characteristics, its consumption has traditionally been limited in at-risk populations, due to its high salt content [2]. High blood levels of sodium cause greater renal perfusion, increasing the excretion of sodium (Na^+^) and water. This compensatory mechanism produces normal blood pressure but is progressively depleted, losing self-regulation. Therefore, high sodium intakes predispose hypertension and the appearance of cardiovascular diseases [3].

The increase in sodium consumption and the need to reduce the prevalence of associated diseases has caused the food industry to develop several methods to reduce the sodium added to products [4]. However, salt is an essential ingredient in meat products because it is responsible for the decrease in water activity, delaying and inhibiting microbial growth, and prolonging their useful life. In addition, salt influences the final texture of the products and provides a characteristic flavor that differentiates them from others [5]. The reduction of salt in dry-cured ham can have direct and indirect consequences, as the biochemical reactions that occur during the ripening period are affected. Salt influences the behavior of the muscular proteolytic system by acting on enzymes such as cathepsins and calpains. These play a very important role during ripening, since they contribute to the appearance of free amino acids and peptides responsible for developing the aroma, smell, and texture of the dry-cured ham [6]. An excessive hydrolysis of proteins by cathepsin B and B + L have been described as responsible for the softness and adhesiveness in the texture profile and the bitter taste of dry-cured ham [7,8]. Furthermore, the sensory study data of the acceptance of hams and the presence of some amino acid derivatives and small peptides have been correlated as components to distinguish defective and normal hams. [9].

Among the strategies for salt reduction, the most studied has been the total or partial substitution of NaCl with other salts. With dry-cured ham obtained from white pig crosses, Frye et al. [10] described the partial reduction of NaCl, by 50% or less, replaced with KCl is possible while maintaining acceptable physical–chemical and sensory attributes [11]. However, other studies reported that although salted dry-cured hams with partially replaced NaCl for KCl were well evaluated, a bitter taste was detected due to potassium [12,13]. Magnesium salts have also been evaluated, but some observed undesirable flavors appeared in the product and had to use other ingredients to mask those flavors [14]. Furthermore, Seong et al. [15] described the possibility of using natural halophytes in different types of dry-cured hams as salt substitutes, obtaining good physical–chemical results. The possibility of using flavor enhancers as substitutes for salt has also been described in other derived meat products [16]. The use of ultrasound at the time of salting [17] or the use of fresh shaped hams are other technological strategies studied to reduce the salt content. Using fresh shaped hams as a raw material would increase the absorption and the diffusion phenomenon of NaCl because of the greater contact surface, producing dry-cured hams with a reduced salt content [18].

To declare that food has reduced sodium, according to European legislation [19], the reduction must be at least 25% compared to a similar product. This reduction is much more complex in Iberian dry-cured hams because they have a lower level of salt per se than dry-cured hams from white commercially crossed pigs. The higher fat content of Iberian dry-cured ham also makes the reduction of salt in this product more complex because fat complicates the diffusion of salt through the tissues, resulting in a delay in the balance of salt inside the ham in the early stages of ripening [20]; in addition, fat could limit the perception of salinity [21,22]. Furthermore, it has been reported that aroma and flavor decrease with higher fat content [23]. Finally, the possibility that dry-cured ham cured using salt substitutes could require a longer processing time in the early stages should be considered [24].

Despite the limitations stated, there is a scarcity of reports that assess the effect of salt reduction on Iberian dry-cured hams. It is expected that using fresh deboned hams may improve the salt diffusion, reducing the salting time and therefore reducing the salt content of the final product. No study has been found on whether deboning the ham would reduce the salt in the product. Therefore, this work aimed to evaluate the effect of salt reduction on the physicochemical composition, free amino acids, and sensory quality of a reduced salt dry-cured boneless ham from an Iberian pig manufactured using a new process. Furthermore, the effect of breed was analyzed using the same parameters of a commercially crossed white pig, to compare with the Iberian product.

## 2. Materials and Methods

### 2.1. Dry-Cured Ham Preparation and Sample Collection

A total of 54 dry-cured hams were used to carry out this study. A batch of Iberian dry-cured hams (RIB) and a batch of dry-cured hams from white commercially crossed pigs (RWC) were produced and supplied by the Spanish meat industry. Fresh raw materials were boned and salted using nitrifying salts and sea salt at a rate of 0.8 days/kg, in a chamber at 3 °C.

After salting, pieces were washed with water, and a traditional curing process was conducted. The resting or postsalting stage started at 3 °C, and the temperature gradually increased to 6 °C. This phase was completed when the hams achieved 18% weight loss. Subsequently, the temperature was increased to 28 °C. The process was concluded when the pieces achieved a loss of 38% of weight. Weight loss was determined by weighing each sample in triplicate in each of the processing stages. The results were expressed as a percentage of weight loss, considering the fresh weight of each piece.

Finally, 18 dry-cured hams were selected from each batch (36 hams). Samples were taken in the following stages: I: Raw muscle; II: Start of post-salting; III: End of postsalting; IV: Drying stage (33% of weight loss); V: Final product (38% of weight loss). To maintain the integrity of the piece throughout processing, samples were taken using a 2 cm diameter stainless steel cylinder, in the area corresponding to the biceps femoris muscle.

Eighteen pieces of Iberian dry-cured hams were manufactured following the traditional curing process as a control product (TIB) (the time of salting stage was 1 day/kg). TIB dry-cured hams were processed with bone.

### 2.2. Physicochemical and Microbial Analyses

Each sample was analyzed in triplicate for each type of dry-cured ham in each of the physicochemical analyses.

The moisture content was determined by drying 5 g of sample at 105 °C for 24 h following the gravimetric procedure described in the ISO 1442 standard [25]. The method of Folch [26] was followed to obtain the intramuscular fat values of the dry-cured ham samples. The ash content was determined using gravimetry, following the AOAC method 920.153 [27].

For the analysis of dry-cured ham salt content, the Volhard method of the ISO 1841-1 standard [28] was modified, adding 2 mL of nitrobenzene to the sample after mineralization with nitric acid and simultaneous oxidation with potassium permanganate, to eliminate settling phases.

Total nitrogen (TN) and nonprotein nitrogen (NPN) were determined using the Kjeldahl method [29], obtaining the crude protein value by multiplying the TN value by 6.25. The proteolysis index (PI) was calculated as the percentage ratio between NPN and TN [30].

Microbiological counts were conducted on the dry-cured ham, following the protocol of Aaslyng, Vestergaard, and Koch [31] with slight modifications. Briefly, 25 g of each sample was taken in triplicate in the final stage (processing stage V). After serial decimal dilutions in 0.9% saline (Merck 106404, Darmstadt, Germany) enriched with peptone to 0.1% (Merck 1.07214.1000), the samples were seeded in Brain Heart Infusion (BHI) (Oxoid CM1136, Thermo Fisher Scientific, Loughborough, UK)) and All-Purpose Tween (APT) agar (Merck 1.10453.0500) and incubated for 5 days at 20 °C. After incubation, *Listeria monocytogenes* was seeded on Oxford agar (Scharlau Chemie SA, Barcelona, Spain), *Salmonella* on SS-Agar (Sigma, 85640, Buchs (SG), Switzerland), *E. coli* on Levine eosin methylene blue agar (Merck 62087, Darmstadt, Germany), *Staphylococcus* on Baird Parker Agar (Scharlau Chemie S.A., Barcelona, Spain), *Clostridium* on Tryptose Sulphite Cycloserine Agar (TSC) without egg yolk (SGL Lab, Corby, UK), Mesophilic aerobes on PCA (Merck, 1.15363.0500 Darmstadt, Germany), and Enterobacteria on (3M Petrifilm 6421, Madrid, Spain).

Mineral composition of samples was determined following the method of Tejada et al. [32], and the free amino acid content was obtained according to Abellán et al. [33].

### 2.3. Determination of Instrumental Color and Texture Profile Analysis (TPA)

Instrumental color was determined by colorimetry using a colorimeter (HunterLab, Colorflex) and the CIELab system. The results were expressed through the coordinates L*, a*, and b*, representing the luminosity, the red–green index, and the yellow index, respectively, and the values of the saturation parameter (chroma) and hue angle (h*). The results were calculated as the mean values of three measurements.

Instrumental texture was analyzed using a QTS-25 texturometer (Brookfield CNS Farnell, Borehamwood, Hertfordshire, England) equipped with a 25 kg load cell and a 10 mm diameter probe [34]. The software Texture Pro v. 2.1. was used for data analysis.

The muscle biceps femoris was analyzed. It was cut into 10 × 10 × 10 mm parallelepipeds, using three parallelepipeds per sample. The room was maintained at 20 °C for the analyses.

The test was conducted by applying two consecutive cycles at a constant speed of 30 mm/s and subjecting the sample to a 50% compression in a direction perpendicular to the muscle fibers.

The TPA was performed on hardness, deformation according to hardness, adhesiveness, cohesiveness, recoverable deformation, springiness, gumminess, and chewiness.

### 2.4. Sensory Analyses (Consumers’ Test)

The sensory evaluation of the dry-cured ham samples was conducted by an untrained panel. A total of 47 participants (53.2% men and 46.8% women) came voluntarily without having received prior training or information. The age range of the participants was 18 to 52 years. The study was conducted in one session where the TIB, RIB, and RWC samples were evaluated by the panelists using a questionnaire that included hedonic evaluation of the appearance, color, odor, texture, salty taste, global taste, and global acceptance. Each attribute was scored by assigning a numerical value through a verbal hedonic scale between 1 (I dislike very much) and 5 (I like very much). In addition, a preference study was also conducted.

The samples used for the sensory consumer test were complete slices of a 1 mm thick cross section of the piece, following the method established in the standards UNE-ISO 6658:2019 [35] and UNE-ISO 4121:2006 [36] and were codified with a random three-digit number.

The results were obtained by calculating the average score given to each attribute of the product evaluated by consumers. To determine the preference between the three samples, the percentage values of choice of the consumer panel were obtained in each type of dry-cured ham studied.

### 2.5. Statistical Analysis

The effect of the processing stage and the effect of breed on physicochemical parameters, salt content, and mineral composition were obtained by a two-way analysis of variance (ANOVA). When the effect of the processing stage or breed was significant (*p <* 0.05), the results were compared using the Fisher LSD test.

To assess if salt reduction and deboning affect the free amino acid content and the sensory quality of Iberian dry-cured ham, a one-way ANOVA was performed. Furthermore, the effect of breed on free amino acid content and sensory quality was studied in salt-reduced dry-cured hams from Iberian and white commercially crossed pigs.

## 3. Results and Discussion

### 3.1. Effect of Processing Stage and Breed on Physicochemical Parameters, Salt Content, Microbial Analyses, and Mineral Composition

Table 1 shows the evolution of the physicochemical parameters and salt content of reduced boneless dry-cured ham obtained from commercial crosses of Iberian and white pigs at different processing stages.

The processing stage affected all physicochemical parameters studied in boneless dry-cured ham of different breeds.

The moisture content in the dry-cured RIB ham was significantly reduced (*p ≤* 0.001), mainly between the drying stage and the final product, whereas the intramuscular fat content increased (*p ≤* 0.05), as observed in the protein values. In this parameter, the increase was more marked between the raw muscle and the drying stage, without observing significant differences between the final product (stage V) and the previous processing stages (*p ≥* 0.05); this was observed in both studied breeds. A similar evolution was observed in the NPN, with significantly increased values mainly between the end of postsalting (stage III) and the end of processing (stage V) (*p ≤* 0.001) in RIB and RWC. Consequently, PI also increased significantly during processing (*p ≤* 0.001). The increase in proteolytic activity of dry-cured hams during their ripening stage (stages IV and V) has been related to the rise in temperature that takes place at these manufacturing stages, resulting in a greater activity of proteolytic enzymes [37]. Physicochemical modifications were in line with what is observed in other dry-cured hams throughout processing [38,39].

NaCl and ash concentrations increased significantly, mainly at the start of postsalting stage due to the incorporation of curing salts associated with the manufacturing process (*p ≤* 0.001 in both cases). A marked increase was observed in the NaCl values of both breeds between stages I (raw muscle) and II (start of postsalting), as well as between the end of postsalting and the last two phases of the production process, due to the decrease in moisture, which takes place during the last stages of production. This phenomenon was also observed in the ash concentration of RWC dry-cured ham (Table 1), which reached its maximum values in the last two stages of the production process, whereas the ash values of the RIB dry-cured ham remained stable between the start of the postsalting stage (stage II) and the final product (stage V). Correct diffusion of salt (NaCl) was achieved with the processing method, with the concentration in dry-cured RWC of 3.77% and 2.86% in the RIB final product (processing stage V). Correct diffusion of salt during processing is important because of the role this ingredient plays. It will not only influence the texture, flavor, and aroma of the product, but will also ensure an optimal microbiological quality [40].

Regarding breed, moisture (*p ≤* 0.001), fat (*p ≤* 0.001), protein (*p ≤* 0.001), TN (*p ≤* 0.001), NPN (*p ≤* 0.001), PI (*p ≤* 0.05), NaCl (*p ≤* 0.05), and ash (*p ≤* 0.001) were also affected.

Moisture values and protein content were significantly higher in dry-cured RWC ham (*p ≤* 0.001), in all stages of processing, in contrast to the fat values, in which the RIB dry-cured ham presented more than double the fat content of RWC (*p ≤* 0.001), due to the high adipogenicity of native pig breeds [41]. These differences between breeds in fat and protein content were like those observed by Lorido et al. [23].

NPN values were significantly higher in RWC dry-cured ham (*p ≤* 0.001) throughout the manufacturing process. As expected, the PI was significantly lower in RIB (*p ≤* 0.05), due to the higher calpains and cathepsins activity in the dry-cured hams of commercially crossed white pigs [42]. In contrast, Córdoba et al. [43] described that proteolysis in Iberian hams could be higher than other types of dry-cured hams, such as Parma ham, due to the longer ripening time and the higher temperatures reached during the process. The PI in both breeds was lower than values reported by Schivazzapa and Virgili [44] in reduced salt Italian dry-cured hams obtained from pig crosses of Large White, Landrace, and Duroc.

The PI observed in the TIB final product (18.58%; value not included in Table 1), was slightly lower than RIB and was like those described in other dry-cured hams [45].

An adequate control of the ripening phase of reduced salt dry-cured hams is essential to assess the prolongation of this processing stage, to avoid texture or aroma problems [46], because free amino acids—resulting from the intense proteolysis the product undergoes during manufacturing—will contribute greatly to developing the sensory characteristics of the final product [47].

NaCl and ash concentrations were also higher in RWC dry-cured ham (*p ≤* 0.001) for all the processing stages. Consistent with salt reduction, the NaCl content in RIB and RWC was less than the values reported by some authors for dry-cured ham from commercial crosses of Croatian white pigs (between 5.76% and 7.01%) [48] and other native breeds (5.75%) [49] for the final product (processing stage V).

The average NaCl concentration was 4.11% in the traditionally manufactured Iberian dry-cured hams, thus a reduction of 30% compared to RIB. In RWC dry-cured hams, a reduction of 27.5% was reached, with an average value considered of 5.20% in traditional white dry-cured ham [50]. According to the Annex of Regulation (EC) No 1924/2006 [19], the claim ‘Reduced in salt’ could be included in the labeling of these products, because the indicated 25% reduction has been exceeded. Pinna et al. [51] also achieved a considerable reduction in salt content in typical Italian dry-cured hams by modifying salt added and the salting time.

Despite reducing the salt content of dry-cured hams, it does not compromise the stability and safety of the final product, as they meet the required microbiological conditions established. In this way, the counts of *Listeria monocytogenes* (in 25 g), *Salmonella* (in 25 g), *E. coli* (cfu/g), *Staphylococcus* (cfu/g), *Clostridium* (cfu/g), Mesophilic aerobes (cfu/g), and Enterobacteria (cfu/g) complied with the limits established by Regulation (EC) No 2073/2005 [52], relative to the microbiological criteria applicable to food products.

Table 2 includes the evolution of the mineral composition of reduced boneless dry-cured ham obtained from commercial crosses of Iberian and white pigs throughout processing.

The processing stage (*p* ≤ 0.001) and breed (*p* ≤ 0.001) significantly modified the concentration of Na, K, Mg, and P. The amount of Zn was only significantly affected (*p ≤* 0.001) by the processing stage, showing higher values in the final stages. Na values increased from the beginning of postsalting (processing stage II) and were always higher in RWC than in RIB, as was observed for NaCl (Table 1). The concentration of the rest of the minerals did not change significantly (*p >* 0.05). The concentration of K, Mg, P, Fe, and Zn was higher than observed in a previous study [53], whereas Na and Mn were lower. Ca values were similar; however, no references could be found for B and Cu.

### 3.2. Free Amino Acids

Results for the effect of Iberian dry-cured ham type and the effect of breed on free amino acid (FAA) content in the final product (processing stage V) are shown in Table 3.

None of the FAA studied was affected by the processing of different Iberian dry-cured ham (traditional and reduced salt boneless manufactured) (*p >* 0.05). The same was observed regarding the total FFA (*p >* 0.05). Therefore, peptidase activity was not significantly affected (*p >* 0.05) by salt reduction (30%) although total FAA content was slightly higher in RIB. These findings disagreed with the study by Cittadini et al. [54], in which an increase in proteolytic phenomenon was observed as the NaCl content decreased.

Abellán et al. [55] described that the concentration of amino acids reflects the proteolysis achieved during the ripening stage of dry-cured meat products. Considering the data obtained in PI, NPN (Table 1), and the total FFA (Table 3), the proteolysis of dry-cured Iberian ham followed an expected evolution, despite the reduction in salt content. According to Lorenzo et al. [56], the proteolytic reactions are promoted in meat products with a partial replacement of sodium.

The differences in amino acid concentration of boneless dry-cured ham of different breeds were not significant (*p >* 0.05). Krvavica et al. [57] also found no effect of breed on the FFA content in two types of dry-cured Croatian ham obtained from different genotypes of commercial white pig crosses.

The differences in the PI between the two breeds studied (Table 1) could be explained by a higher protease activity (calpain and cathepsins) of RWC dry-cured hams. However, no differences in FAA concentration were observed, probably because the breed did not affect peptidases activity.

### 3.3. Instrumental Color and Texture Profile

Table 4 shows the effect Iberian dry-cured ham type and the effect of breed on the instrumental color.

The instrumental color parameters were not affected by different processes for Iberian dry-cured ham (*p >* 0.05 in all cases), agreeing with the data obtained by Lorenzo et al. [58], who studied the effect of partial replacement of NaCl with other salts on dry-cured *lacón*. In contrast, Tejada et al. [32] observed an effect of different salt formulation on the color parameters of another derived meat pig product (Spanish *chorizo*) describing a higher luminosity value (L*) in the product cured with a traditional formulation (with NaCl as the main ingredient), as well as higher values of b*, C*, and h*. No breed effect on color parameters was observed (*p >* 0.05 in all cases). The range of L*, a*, and b* values obtained in the two types of Iberian dry-cured ham studied (TIB and RIB) and in the reduced salt dry-cured ham, obtained from white commercial pig crosses, were higher than dry-cured ham obtained from the Celta pig by Bermúdez et al. [38].

Table 5 shows the effect Iberian dry-cured ham type and breed on the instrumental texture.

The processing of Iberian dry-cured ham and the breed had a significant effect on C1 hardness, C2 cohesiveness, C2 gumminess, C2 chewiness, and C2 hardness (Table 5). All cited texture profile parameters were higher in RIB compared to TIB. Despite the differences observed in the TPA, consumers did not report an effect of processing between the two Iberian dry-cured hams studied (Table 6). Although the TPA confirmed the differences found in the sensory analysis regarding breed, where consumers scored RIB texture better than RWC’s (Table 6).

The data here are partially consistent with the study of Tejada et al. [32], in which the authors found an effect of salt reduction on hardness, cohesiveness, gumminess, and chewiness in another derived meat pig product. However, TPA hardness values where higher in the NaCl reduced product of the cited study, contrary to the data obtained in this study, as RIB showed higher values. The authors associated this phenomenon with the inhibition of cathepsins by NaCl, which reduces the proteolysis activity affecting the texture of the product. Therefore, we can conclude that proteolysis of RIB was not affected significantly, considering TPA hardness values (Table 5) and according to data obtained for the FFA (Table 3). Regarding breed, RIB’s higher hardness values could be related to the lower moisture content obtained than the RWC dry-cured ham (Table 1).

Cittadini et al. [54] also studied the effect of NaCl replacement by other chloride salts on TPA parameters (hardness, springiness, cohesiveness, gumminess, and chewiness) of foal Cecina, a similar dry-cured product, but in contrast to our result, they only found significant differences in the springiness.

### 3.4. Consumer Sensory Acceptability and Preference

Table 6 shows the acceptability scores for TIB, RWC, and RIB dry-cured hams given by the consumer panel.

Regarding the type of processing, the panel of consumers scored all the attributes in RIB dry-cured ham higher than the TIB dry-cured ham, although none were significantly different (*p >* 0.05). Therefore, the consumer acceptability of sensory traits is similar in both types of dry-cured ham; thus, the reduction of the salt content and the deboning do not affect the organoleptic characteristics of the Iberian dry-cured ham. The results agree with other studies of cured meat in which the palatability and texture were not compromised with the reduction of salt in respect to the traditional method [59]. However, contrasting results have been reported in cooked ham products, where texture, flavor, and overall consumer acceptability were significantly affected by salt reduction [60].

In Italian dry-cured ham obtained from white commercial pig crosses, a greater acceptability was observed in salt-reduced dry-cured ham in respect to the same product processed after traditional manufacturing [44].

Both types of Iberian ham (TIB and RIB) obtained global acceptance scores above 4 (I like) on the scale of 1–5, so it can be asserted that the consumer accepts Iberian dry-cured ham more.

Regarding the effect of the breed on the consumer acceptability of dry-cured ham, all sensory traits were scored significantly high by the untrained panel in dry-cured RIB ham compared to dry-cured RWC ham (*p ≤* 0.05).

The intramuscular fat content of the product has been described among the main composition parameters that influence the organoleptic quality in dry-cured products [61]. As has been described, this parameter was significantly higher in RIB dry-cured ham compared to RWC dry-cured ham, thus data agreed with other studies on Iberian pig dry-cured products [62] and other native breeds [63], where it was observed that products obtained from autochthonous breeds were better valued by consumers.

In the preference study, 6.38% of consumers preferred the RWC dry-cured ham, 63.83% the RIB dry-cured ham, and 29.79% the TIB. Therefore, from a consumer viewpoint, the reduction of salt in dry-cured ham improved the perception.

Regarding the preference between the two Iberian products, 70% of the consumers chose the reduced salt product, although no significant differences were detected in the evaluation of the acceptance of the salty taste (Table 6).

## 4. Conclusions

The deboning of the dry-cured ham before processing and the reduction of the salting time produced dry-cured hams with lower salt content. A greater reduction in salt was achieved in Iberian dry-cured ham than in that from white commercial pig crosses due to the different physicochemical and biochemical characteristics of its meat. The decreased salt concentration achieved allows the inclusion of the nutritional claim “reduced sodium/salt content” compared to similar products.

The reduction in salt and deboning did not have a negative effect on the proteolysis of dry-cured ham (both Iberian and white commercial pig crosses), as no differences in FFA content were observed. Furthermore, the PI increased only slightly (which was higher in dry-cured hams from white commercial pig crosses).

Reduced salt dry-cured hams have adequate consumer acceptance, adequate instrumental color, and texture characteristics. Although the processes modified the texture of the reduced Iberian dry-cured hams, their sensory characteristics were better valued than those with bone and following the traditional manufacturing processes.

## Figures and Tables

**Table 1:** Effect of processing stage and breed on the physicochemical parameters and salt content of boneless dry-cured ham. Results are expressed in g/100 g of product (means values ± SEM).

		Processing Stage				*p*-Value			
		I	II	III	IV	V	Processing Stage	Breed	Interaction
Moisture	RIB	53.12 ± 1.31 ^a^	52.82 ± 1.77 ^a^	47.12 ± 2.11 ^a^	37.35 ± 1.83 ^b^	31.59 ± 1.26 ^b^	0.000	0.000	0.000
	RWC	57.82 ± 6.93 ^a^	56.65 ± 1.58 ^a^	53.49 ± 1.71 ^a^	52.08 ± 1.29 ^a^	51.46 ± 0.64 ^a^			
Fat	RIB	25.00 ± 1.43 ^bc^	21.10 ± 2.32 ^abc^	22.77 ± 2.37 ^abc^	27.42 ± 2.00 ^cd^	33.87 ± 1.94 ^d^	0.028	0.000	0.007
	RWC	17.34 ± 4.54 ^abc^	16.18 ± 1.00 ^ab^	17.61 ± 1.43 ^ab^	16.72 ± 1.01 ^ab^	15.99 ± 0.46 ^a^			
Protein	RIB	15.00 ± 0.35 ^b^	16.48 ± 0.90 ^ab^	19.53 ± 0.30 ^ac^	22.32 ± 0.73 ^c^	22.13 ± 1.19 ^c^	0.000	0.000	0.001
	RWC	16.10 ± 1.53 ^ab^	17.92 ± 0.10 ^ab^	20.43 ± 0.50 ^ac^	28.31 ± 0.55 ^d^	29.68 ± 0.52 ^d^			
Total Nitrogen	RIB	2.40 ± 0.06 ^b^	2.63 ± 0.14 ^ab^	3.13 ± 0.05 ^ac^	3.57 ± 0.12 ^c^	3.54 ± 0.19 ^c^	0.000	0.000	0.001
	RWC	2.57 ± 0.25 ^ab^	2.87 ± 0.02 ^ab^	3.27 ± 0.08 ^ac^	4.53 ± 0.09 ^d^	4.75 ± 0.08 ^d^			
Nonprotein Nitrogen	RIB	0.25 ± 0.06 ^a^	0.18 ± 0.07 ^a^	0.30 ± 0.07 ^a^	0.48 ± 0.31 ^ab^	0.74 ± 0.16 ^bc^	0.000	0.000	0.001
	RWC	0.34 ± 0.09 ^ab^	0.26 ± 0.11 ^a^	0.33 ± 0.13 ^a^	1.25 ± 0.42 ^d^	1.04 ± 0.18 ^cd^			
Proteolysis Index	RIB	10.54 ± 0.86 ^a^	6.73 ± 1.42 ^a^	9.50 ± 1.10 ^a^	13.88 ± 3.62 ^ab^	20.90 ± 1.24 ^bc^	0.000	0.012	0.071
	RWC	13.45 ± 2.24 ^abc^	9.01 ± 1.87 ^a^	10.07 ± 1.97 ^a^	27.85 ± 5.82 ^c^	22.02 ± 1.59 ^bc^			
NaCl	RIB	0.51 ± 0.27 ^d^	1.96 ± 0.69 ^a^	1.99 ± 0.17 ^a^	2.79 ± 0.14 ^abc^	2.86 ± 0.21 ^abc^	0.000	0.016	0.176
	RWC	0.16 ± 0.02 ^d^	2.33 ± 0.08 ^ab^	2.43 ± 0.13 ^ab^	3.64 ± 0.18 ^bc^	3.77 ± 0.20 ^c^			
Ash	RIB	2.21 ± 0.51 ^c^	5.58 ± 1.65 ^abc^	5.59 ± 0.68º ^abc^	5.44 ± 0.48 ^ab^	5.68 ± 0.85 ^ab^	0.000	0.000	0.012
	RWC	2.39 ± 0.44 ^bc^	8.81 ± 0.88 ^ad^	6.84 ± 0.61 ^ad^	10.92 ± 0.85 ^d^	10.41 ± 0.40 ^d^			

Two-way ANOVA. ^a,b,c,d^ Values within a row with different superscripts differ significantly at *p ≤* 0.05 (Fisher LSD Test). SEM: standard error of the mean; RIB: reduced Iberian dry-cured ham; RWC: reduced dry-cured ham from white commercial pig crosses; I: Raw muscle; II: Start of postsalting; III: End of postsalting; IV: Drying stage; V: Final product.

**Table 2:** Effect of the processing stage and the breed on the mineral composition of boneless dry-cured ham.

	Processing Stage						*p*-Value			RDA
		I	II	III	IV	V	Processing Stage	Breed	Interaction	
Na ^1^	RIB	0.23 ± 0.08 ^a^	0.83 ± 0.32 ^b^	0.81 ± 0.11 ^b^	1.01 ± 0.11 ^bc^	1.13 ± 0.14 ^c^	0.000	0.000	0.072	0.006
	RWC	0.15 ± 0.00 ^a^	1.24 ± 0.14 ^c^	1.16 ± 0.11 ^c^	1.75 ± 0.17 ^de^	1.54 ± 0.08 ^d^				
K ^1^	RIB	0.23 ± 0.04 ^a^	0.28 ± 0.03 ^a^	0.30 ± 0.02 ^a^	0.33 ± 0.03 ^a^	0.45 ± 0.03 ^ab^	0.000	0.000	0.000	2000
	RWC	0.28 ± 0.05 ^a^	0.34 ± 0.01 ^a^	0.39 ± 0.02 ^a^	0.96 ± 0.04 ^b^	0.64 ± 0.02 ^b^				
Ca ^1^	RIB	0.02 ± 0.01 ^a^	0.01 ± 0.00 ^a^	0.02 ± 0.00 ^a^	0.01 ± 0.00 ^a^	0.01 ± 0.00 ^a^	0.536	0.673	0.675	800
	RWC	0.01 ± 0.00 ^a^	0.01 ± 0.00 ^a^	0.02 ± 0.00 ^a^	0.01 ± 0.00 ^a^	0.02 ± 0.01 ^a^				
Mg ^1^	RIB	0.02 ± 0.00 ^a^	0.02 ± 0.00 ^a^	0.02 ± 0.00 ^a^	0.02 ± 0.00 ^a^	0.03 ± 0.00 ^ab^	0.000	0.001	0.061	375
	RWC	0.02 ± 0.00 ^a^	0.02 ± 0.00 ^a^	0.03 ± 0.00 ^a^	0.04 ± 0.00 ^b^	0.04 ± 0.00 ^b^				
P ^1^	RIB	0.15 ± 0.05 ^a^	0.16 ± 0.01 ^a^	0.18 ± 0.01 ^a^	0.20 ± 0.01 ^a^	0.23 ± 0.02 ^a^	0.000	0.000	0.000	700
	RWC	0.16 ± 0.03 ^a^	0.19 ± 0.01 ^a^	0.23 ± 0.01 ^a^	0.34 ± 0.01 ^b^	0.31 ± 0.01 ^b^				
Fe ^2^	RIB	9.66 ± 0.56 ^a^	6.75 ± 0.80 ^a^	5.69 ± 1.05 ^a^	14.13 ± 5.48 ^a^	11.66 ± 1.03 ^a^	0.133	0.737	0.978	14
	RWC	8.06 ± 1.68 ^a^	7.75 ± 0.60 ^a^	8.33 ± 1.60 ^a^	14.26 ± 2.78 ^a^	13.02 ± 3.83 ^a^				
Cu ^2^	RIB	1.39 ± 0.57 ^a^	1.06 ± 0.26 ^a^	0.70 ± 0.14 ^a^	3.86 ± 1.96 ^a^	1.5 ± 0.27 ^a^	0.577	0.537	0.509	1
	RWC	0.32 ± 0.12 ^a^	1.62 ± 0.69 ^a^	1.41 ± 0.14 ^a^	1.24 ± 0.59 ^a^	1.79 ± 0.23 ^a^				
Mn ^2^	RIB	0.62 ± 0.38 ^a^	0.15 ± 0.02 ^a^	0.27 ± 0.04 ^a^	0.29 ± 0.05 ^a^	0.10 ± 0.03 ^a^	0.478	0.541	0.673	2
	RWC	0.31 ± 0.17 ^a^	0.21 ± 0.08 ^a^	0.20 ± 0.04 ^a^	0.12 ± 0.05 ^a^	0.26 ± 0.011 ^a^				
Zn ^2^	RIB	21.64 ± 1.80 ^ab^	15.91 ± 7.80 ^a^	16.56 ± 2.42 ^a^	21.53 ± 2.70 ^ab^	24.59 ± 1.74 ^bc^	0.000	0.169	0.251	10
	RWC	16.47 ± 3.22 ^a^	19.76 ± 2.53 ^a^	18.83 ± 2.44 ^a^	26.69 ± 1.58 ^bc^	29.76 ± 2.90 ^c^				
B ^2^	RIB	0.78 ± 0.53 ^a^	0.36 ± 0.06 ^a^	0.18 ± 0.06 ^a^	0.34 ± 0.06 ^a^	0.47 ± 0.08 ^a^	0.882	0.538	0.598	Not declared
	RWC	0.14 ± 0.06 ^a^	0.21 ± 0.06 ^a^	0.36 ± 0.01 ^a^	0.38 ± 0.19 ^a^	0.51 ± 0.15 ^a^				

Two-way ANOVA. ^a,b,c,d,e^ Values within a row with different superscripts differ significantly at *p ≤* 0.05 (Fisher LSD Test). ^1^ Results are expressed in g/100 g of product (means values ± standard error of mean). ^2^ Results are expressed in mg/100 g of product (means values ± standard error of mean). RIB: reduced Iberian dry-cured ham; RWC: reduced dry-cured ham from white commercial pig crosses; I: Raw muscle; II: Start of post-salting; III: End of post-salting; IV: Drying stage; V: Final product; RDA: Recommended Daily Allowances of minerals.

**Table 3:** Effect of Iberian dry-cured ham type (processing) and effect of breed on free amino acid content (FAA). The results are expressed in g/kg of dry matter as mean values ± SEM.

FAA	Dry-Cured Ham Type			*p*-Value	
	TIB	RIB	RWC	Processing	Breed
Asp	2.08 ± 0.06	2.05 ± 0.54	1.94 ± 0.74	0.962	0.921
Glu	3.98 ± 0.11	4.26 ± 1.04	4.87 ± 0.92	0.809	0.706
Ser	1.51 ± 0.05	1.34 ± 0.21	1.83 ± 0.47	0.530	0.443
His	0.91 ± 0.02	1.00 ± 0.25	1.17 ± 0.25	0.760	0.677
Gly	1.23 ± 0.01	1.42 ± 0.40	1.79 ± 0.37	0.685	0.566
Thr	1.45 ± 0.03	1.32 ± 0.28	1.81 ± 0.30	0.685	0.352
Arg	1.75 ± 0.16	1.06 ± 0.02	1.32 ± 0.22	0.051	0.368
Ala	4.02 ± 0.14	4.70 ± 1.06	5.67 ± 0.46	0.590	0.489
Tyr	0.70 ± 0.01	0.89 ± 0.16	1.44 ± 0.47	0.377	0.384
Cys	nd	nd	nd	-	-
Val	2.10 ± 0.05	2.22 ± 0.53	2.51 ± 0.53	0.847	0.732
Met	0.53 ± 0.21	0.64 ± 0.23	0.79 ± 0.01	0.764	0.580
Phe	1.42 ± 0.15	1.56 ± 0.38	1.82 ± 0.35	0.765	0.669
Ile	1.48 ± 0.09	1.64 ± 0.44	1.90 ± 0.42	0.760	0.706
Leu	2.43 ± 0.19	2.65 ± 0.71	3.21 ± 0.79	0.786	0.654
Lys	3.73 ± 0.10	4.13 ± 1.15	5.00 ± 1.17	0.766	0.649
Pro	1.87 ± 0.01	1.96 ± 0.40	2.18 ± 0.25	0.837	0.688
Total FAA	31.19 ± 0.38	32.84 ± 7.76	39.26 ± 7.71	0.852	0.617

TIB: traditional Iberian dry-cured ham; RIB: reduced Iberian dry-cured ham; RWC: reduced dry-cured ham from white commercial pig crosses; SEM: standard error of the mean; *p*-value Processing: One-way ANOVA between TIB and RIB; *p*-value Breed: One-way ANOVA between RIB and RWC (*p*-value significant at *p ≤* 0.05); nd: nondetected.

**Table 4:** Effect of Iberian dry-cured ham type (processing) and effect of breed on instrumental color. Results are expressed as mean values ± SEM.

Color Parameter	Dry-Cured Hams			*p*-Value	
	TIB	RIB	RWC	Processing	Breed
Lightness (L*)	47.22 ± 4.23	53.03 ± 1.79	57.88 ± 0.85	0.165	0.164
Redness (a*)	24.70 ± 2.96	23.36 ± 1.62	20.72 ± 1.06	0.692	0.392
Yellowness (b*)	27.34 ± 5.26	28.46 ± 1.24	31.09 ± 3.19	0.758	0.363
Chroma (C*)	37.26 ± 4.61	37.09 ± 1.30	37.47 ± 2.70	0.961	0.891
Hue angle (h◦)	47.01 ± 6.44	50.75 ± 2.52	55.99 ± 3.04	0.519	0.298

TIB: traditional Iberian dry-cured ham; RIB: reduced Iberian dry-cured ham; RWC: reduced dry-cured ham from white commercial pig crosses; SEM: standard error of the mean.

**Table 5:** Effect of Iberian dry-cured ham type (processing) and effect of breed on instrumental texture. Results are expressed as mean values ± SEM.

Texture Parameter	Dry-Cured Hams			*p*-Value	
	TIB	RIB	RWC	Processing	Breed
C1 Hardness (N)	6.89 ± 0.95	8.61 ± 0.31	5.84 ± 0.54	0.041	0.000
C1 Hardness deformation	2.95 ± 0.07	2.97 ± 0.00	2.93 ± 0.05	0.684	0.179
C1 Adhesiveness (mJ)	0.72 ± 0.09	1.03 ± 0.39	0.57 ± 0.09	0.249	0.121
C2 Cohesiveness	6.10 ± 0.74	7.93 ± 0.38	4.99 ± 0.03	0.021	0.000
C2 Recoverable deformation (mm)	0.53 ± 0.05	0.60 ± 0.03	0.56 ± 0.02	0.091	0.152
C2 Springiness	0.95 ± 0.19	1.14 ± 0.10	1.10 ± 0.01	0.122	0.558
C2 Gumminess (N)	1.56 ± 0.03	2.12 ± 0.08	1.81 ± 0.04	0.000	0.000
C2 Chewiness (mJ)	3.11 ± 0.36	5.16 ± 0.39	3.59 ± 0.60	0.000	0.023
C2 Hardness (N)	5.42 ± 0.90	10.97 ± 0.87	6.03 ± 0.23	0.001	0.000

TIB: traditional Iberian dry-cured ham; RIB: reduced Iberian dry-cured ham; RWC: reduced dry-cured ham from white commercial pig crosses; SEM: standard error of the mean; N: Newton; mJ: millijoules; mm: millimeters; *p*-value Processing: One-way ANOVA between TIB and RIB; *p*-value Breed: One-way ANOVA between RIB and RWC (*p*-value significant at *p ≤* 0.05).

**Table 6:** Effect of Iberian dry-cured ham type (processing) and effect of breed on consumer acceptability by a consumer panel. Results are expressed as mean values ± SEM *.

	Dry-Cured Ham Type			*p*-Value	
	TIB	RIB	RWC	Processing	Breed
Appearance	3.66 ± 0.13	4.17 ± 0.15	2.62 ± 0.16	0.061	0.006
Color	3.83 ± 0.12	4.13 ± 0.13	2.68 ± 0.15	0.159	0.021
Odor	3.55 ± 0.15	3.87 ± 0.13	2.89 ± 0.13	0.200	0.008
Texture	3.81 ± 0.11	3.98 ± 0.14	2.83 ± 0.13	0.232	0.013
Salty taste	3.57 ± 0.13	3.87 ± 0.13	2.87 ± 0.13	0.252	0.001
Global taste	3.96 ± 0.12	4.06 ± 0.10	2.81 ± 0.14	0.224	0.002
Global acceptance	4.00 ± 0.12	4.09 ± 0.12	2.57 ± 0.123	0.220	0.005

* Attributes were scored assigning a numerical value through a verbal hedonic scale between 1 (I dislike very much) and 5 (I like very much); SEM: standard error of mean; TIB: traditional Iberian dry-cured ham; RIB: reduced Iberian dry-cured ham; RWC: reduced dry-cured ham from white commercial pig crosses; *p-*value Processing: One-way ANOVA between TIB and RIB; *p*-value Breed: One-way ANOVA between RIB and RWC (*p*-value significant at *p ≤* 0.05).
